# Supplementary material for: Goal-line oracles: Exploring accuracy of wisdom of the crowd for football predictions
Source: PLoS One. 2025 Jan 31;20(1):e0312487. doi: 10.1371/journal.pone.0312487 (PMC11785260; doi:10.1371/journal.pone.0312487)
Supplement: Attachment — Submitted filename: review_WoC_xG_forecast.pdf [file pone.0312487.s001.pdf]

Goal-Line Oracles: A Longitudinal Exploration of the Wisdom of the Crowd for Football Predictions

Referee report

June 14th, 2024

I. General comment

I want to thank the authors for this quality work and the editor for the invitation to review. This work explores the ability of crowds and xG to surpass betting odds to forecast football matches results. The application of wisdom of crowds to match forecasting is a promising topic, and the approach of the authors seems relevant. I am quite positive about this work and, as I will focus more on the issues in the following sections, I want to emphasize that I believe the paper will eventually make it to the publication stage. However, the current form of the paper presents several issues. I hope these comments will help the authors to improve his work.

II. Recommendation

I would recommend major revision to the paper.

III. Revision

Below, I offer my comments, some critical, others as suggestions for added value. Last, I offer my notes on typos and minor issues to be addressed.

Note: If a second round takes place, I would suggest numbering the pages (and maybe the lines) to ease the review. The page numbers below refer to the pages in the proof I received. As a reference, the introduction starts on page 8.

a. Critical issues

My main concerning the manuscript is the relative weakness of the empirical setup, which limits the reach of the results. However, the research question is particularly relevant, and the results are promising. To overcome the empirical issues and especially, the limited sample sizes, I would like to see robustness checks and power/sensitivity analyses (for example, but any suggestion from the author is welcome). What is important to me is that the author shows that the setup does not influence the results: changing the aggregation heuristic, using different tests, etc.

1. Given that the sample sizes are limited, I would like to see some power analysis to strengthen the robustness of the results.
2. On p.18, I would like to know how sensitive the results are to the heuristics used (0.04 gap = draw and >0.5 gap = win)? Would simply compare WoC estimates (if WoCa > WoCb then a win, with a tolerance for draws) yield the same results? Some robustness checks would be helpful to strengthen your results.
3. The authors use mean aggregation strategy to summarise the opinion of the crowd. Why not test other strategies (the one proposed by Navajas and colleagues, median, etc.)? In my opinion, this should not take to much time and computation while enhancing the robustness of the analysis.

Other critical issues are:

4. On p. 16, the author presents "exploratory corrections of predictions" but I do not get the purpose of this operation: if you remove the average difference between the two, you will end up with close mean, this is a logical consequence but does not prove your point in my opinion.
5. From a conceptual perspective, xG is more about chance creation than about goal scored. On the other hand, the WoC assessment is about goal scored (given the formulation of the question asked to participants). Therefore, I think WoC assessment and xG are not directly comparable because chance creation and goal scored are two related yet different issues. Maybe comparing xG and WoC assessment relations to actual goals would be a better idea than comparing WoC assessment to xG directly.
6. Overall, I think that the literature section lacks some important elements:

- On the concept of "wisdom of the crowds" I general, I suggest you refer to Surowiecki (2005)
- On the forecasting of football match results with "human opinion", you may read Brown et al. (2018) who use sentiment analysis from Twitter posts, and Peeters (2018), who use Transfermarkt crowd-sourced values.
- On p.11 the author states "Related to the current study, other papers explore crowd predictions and transfer market valuations (e.g. 12, 13)." Research on Transfermarkt values is the main literature on wisdom of the crowds applied to football and I believe you should spend more time on it. For empirical and conceptual overview I suggest Franceschi et al. (2023a, 2023b).
- The author should spend more time on expected goals and give references (Pollard et al., 2004; Ensum et al., 2005, Stats Perform 2012). They could also spend some time discussing the limit of the xG approach (Spearman, 2018)

7. On p.17, in my opinion, the predicting power of the wisdom of the crowd to predict xG is not so high in terms of R^2. I would like the author to critically reflects on these results. In particular the author says "This supports H1 that Wisdom of the Crowds ExG estimates will not differ from observed ExG", but such a low R^2 is not sufficient to verify this hypothesis in my opinion. It is arguable however and the author should provide further details to support their point. It would be interesting to rely on metrics traditionally used to assess prediction power rather than R^2 (MAE, RMSE, MAPE). I would be interested to see the distribution of "residuals" (difference between WoC assessment and xG), which would be more interesting than average comparison.

b. Suggestions

8. I would suggest using the usual abbreviation for Expected Goals, i.e. xG, not ExG.
9. The study is said to be longitudinal but my understanding is that the participants in the survey can change every week. Why is the study called longitudinal then?
10. Participants are people who listed they interest in the project or saw the link on Twitter and Linkedin. I would like the author to spend more time on the potential biases arising from this sampling.
11. On p.14 the author states "However, as we observe no differences in statistical fit because of the number of participants, this may be less problematic." Please provide evidence in supplementary materials.
12. On p.14 (but elsewhere too) the author uses the terms "observed ExG" (in contrast to "crowdsourced xG"). I would suggest not using "observed" in this context as xG are estimated from a statistical model. Maybe "estimated" or "ex-post" would be better suite. Another (and better in my opinion) solution would be to stop using "xG" when talking about Wisdom of the Crowd assessment. The author would then be able to use xG only for "actual" xG to avoid confusion. This would greatly improve readability.
13. On p.19 why is the hypothetical investment 3600 and not 360 (36*10£)?
14. On p.20 the author states "Our results support all three hypotheses" but only two hypotheses were stated in the introduction.
15. On p.20 the author highlights an outlier participant but was it removed or included anyway?

c. Typos and other clarifications

p. 10: “alongside” → Capitalise -A

p.15 “of vice versa” → “or vice versa”

IV. Bibliography

Brown, A., Rambaccussing, D., Reade, J.J. and Rossi, G. (2018). Forecasting with social media: evidence from tweets on soccer matches. Econ Inq, 56: 1748-1763. https://doi.org/10.1111/ecin.12506

Ensum, J., Pollard, R., & Taylor, S. (2005, May). Applications of logistic regression to shots at goal at asssociation football. In Science and football V: The proceedings of the Fifth World Congress on Science and Football (pp. 214-221).

Franceschi, M., Brocard, J.-F., Follert, F., & Gouguet, J.-J. (2023). Determinants of Football Players' Valuation: A Systematic Review. Journal of Economic Surveys, 38(3), 577–600. https://doi.org/10.1111/joes.12552

Franceschi, M., Brocard, J., Follert, F., & Gouguet, J. (2023). Football players in light of economic value theory: Critical review and conceptualisation. Managerial and Decision Economics, 45(2), 896–920. https://doi.org/10.1002/mde.4039

Peeters, T. (2018). Testing the Wisdom of Crowds in the field: Transfermarkt valuations and international soccer results. International Journal of Forecasting, 34(1), 17–29. https://doi.org/10.1016/j.ijforecast.2017.08.002

Pollard, R., Ensum, J., & Taylor, S. (2004). Estimating the probability of a shot resulting in a goal: The effects of distance, angle and space. International Journal of Soccer and Science, 2(1), 50-55. https://www.researchgate.net/profile/Richard-Pollard-3/publication/240641737_Estimating_the_probability_of_a_shot_resulting_in_a_goal_The_effects_of_distance_angle_and_space/links/5440a87c0cf2be1758d009e9/Estimating-the-probability-of-a-shot-resulting-in-a-goal-The-effects-of-distance-angle-and-space.pdf

Spearman, W. (2018, February). Beyond expected goals. In Proceedings of the 12th MIT sloan sports analytics conference (pp. 1-17).

Stats Perform. (2012). Assessing the performance of Premier League goalscorers. https://www.statsperform.com/resource/assessing-the-performance-of-premier-league-goalscorers/

Surowiecki, J. (2005). The Wisdom of Crowds. Anchor Books.
