# Supplementary material for: Goal-line oracles: Exploring accuracy of wisdom of the crowd for football predictions
Source: PLoS One. 2025 Jan 31;20(1):e0312487. doi: 10.1371/journal.pone.0312487 (PMC11785260; doi:10.1371/journal.pone.0312487)
Supplement: Attachment — Submitted filename: review_PONE_D-24-17410_R1.pdf [file pone.0312487.s004.pdf]

Goal-Line Oracles: Exploring Accuracy of Wisdom of the Crowd for Football Predictions

Referee report – Second round

September 30th, 2024

I. General comment

I thank the author for their answers and for engaging wholeheartedly with the reviews. Most of my points have been addressed and I am happy with the results. My remaining desired revisions concern the readability of the paper which should be improved. Although I try to provide helpful suggestions, I encourage the author to go beyond my comments to improve the overall structure and readability.

II. Recommendation

I would recommend minor revision to the paper.

III. Revision

On p.9 and 10, I would swap the paragraphs starting with "Second, the nature…" and "For each round of matches…" and remove the mention "which is sufficient statistical power as discussed below" as it would be discussed right after.

On p.10, the conclusion to the power analysis is: "sample size of 21 participants is recommended. As the study averages 25.21 participants per round, it suggests that the study was adequately powered.". I believe the author takes a shortcut here: the average may well be 25.21 with some rounds having less than 21 participants, hence being underpowered. As a matter of fact, I checked 3 rounds on OSF (rounds 1, 10 and 12) and found out that the round 12 file contains only 19 rows. In my opinion, this is not a big issue, but I would appreciate a better formulation and more transparency (providing an appendix with the number of participants per round would be great).

On p.16, based on my suggestion, the author details Navajas et al.'s method to derive WoC estimates. I apologize as I understand only now that it is not feasible in the article's empirical context. Therefore, it is not necessary to discuss the method in such details, and I believe that the author should shorten the paragraph and ease the reading. I sincerely apologize for the inconvenience. On the other hand, the section concerning the median is a great addition.

Overall, the author should enhance the readability by clarifying the structure of the paper. Here is a non-exhaustive list of ideas:

- better balance the sections (5 pages for introduction, 2.5 pages for methods, 7.5 for results and 2 for discussion and conclusion) by adding a literature review section which would shorten the introduction and make it more impactful;
- provide an outline at the end of the introduction ;
- develop sharper subtitles in the Results section.

IV. Typos and corrections

On p.13, I think a word is missing before H1: "the results provide tentative support H1 (Wisdom of the Crowds XG estimates will not differ from observed XG)."
