# Supplementary material for: Goal-line oracles: Exploring accuracy of wisdom of the crowd for football predictions
Source: PLoS One. 2025 Jan 31;20(1):e0312487. doi: 10.1371/journal.pone.0312487 (PMC11785260; doi:10.1371/journal.pone.0312487)
Supplement: Attachment — Submitted filename: football forecast report.docx [file pone.0312487.s002.docx]

Report on Wisdom of Crowds and Predicting Football Match Outcomes

The paper asks a natural question, do crowd sourced predictions of football outcome make more accurate predictions than other sources of forecasts. The approach is to use a convenince sample of friends, coworkers and individuals responding to a request on the internet to collect predictions about the number of goals each team will score in each of the matches in the English Premier League during the 2022-2023 season. These predictions are compared to those based on bookmaker odds and generated by a third-party prediction group called Understat. Evidence is that the crowd predictions are not generally different, on average, than those of Understat.

While the question is an interesting one, the methods and results are problematic. First, the crowd size varies substantially from week to week and only 4 individuals participated throughout the full season. More importantly, comparing the predictions of the crowd to the predictions of a third party without any discussion of how that organization makes the predictions raises many questions.

1) Is that organization using a crowd-based method? If it is, the comparison in this paper is between two different crowds, one presumably chosen to be a random sample and one a convenience sample. The random sample could be much larger than the sample in this study, so the comparison is also between predictions from a large crowd versus those from a small crowd. The point is that the nature of the comparison is unclear.
2) Does the organization use an econometric model to forecast goals? If so, what is the nature of this model? What factors does it use? Why is this forecasting group used rather than some other forecast? How would the crowd stack up against those of other predictors of math outcomes?

Some of the discussion is unclear, uninformative or wrong. Of course some forecasts are bigger and others smaller than the expected goals, both are random variables with distributions that overlap but which are, in principle, centered around the same mean. Of course some individual’s forecasts are greater than the mean and some below it. That is the nature of a mean.

The big 6 clubs are not always the favorites. The discussion of favorites seems to suggest they are.

The bottom line for me is that the paper does not provide enough background to convince the reader that the comparison being made is generally informative.
